# Integrated Devices: A New Regulatory Pathway to Promote Revolutionary Innovation

**DOI:** 10.1111/1468-0009.12692

**Published:** 2024-01-22

**Authors:** TED CHO, VRUSHAB GOWDA, HENNING SCHULZRINNE, BRIAN J. MILLER

**Affiliations:** ^1^ University of California San Francisco California USA; ^2^ Massachusetts General Hospital Boston USA; ^3^ Columbia University New York USA; ^4^ The Johns Hopkins University School of Medicine Baltimore USA; ^5^ The Johns Hopkins Carey Business School; ^6^ American Enterprise Institute

**Keywords:** Food and Drug Administration, health technology, medical devices, regulatory policy

## Abstract

**Policy Points**

- Current medical device regulatory frameworks date back half a century and are ill suited for the next generation of medical devices that involve a significant software component.
- Existing Food and Drug Administration efforts are insufficient because of a lack of statutory authority, whereas international examples offer lessons for improving and harmonizing domestic medical device regulatory policy.
- A voluntary alternative pathway built upon two‐stage review with individual component review followed by holistic review for integrated devices would provide regulators with new tools to address a changing medical device marketplace

Virtually all modern medical devices now include embedded software, ranging from basic user interfaces and network connectivity to sophisticated artificial intelligence (AI) and machine learning (ML). Although integrated devices (devices that tightly integrate software components to drive the function of traditional medical devices and create improved, augmented, or new capabilities) have existed for years, the size of this market has grown rapidly, with connected health care devices (devices that send and receive orders from other devices or networks, such as the internet) alone creating a market valued at USD $28.24 billion in 2020 and is expected to reach USD $94.32 billion by 2026 at a compound annual growth rate of 18.92%.^1^, ^2^ The prevalence of these integrated devices is blurring the lines between previously distinct categories, such as hardware and software, medical devices and clinical informatics, and regulated medical devices and off‐the‐shelf third‐party software. Multiple product areas are now converging and are often even connected with one another, linked across mobile, home internet, or local area networks in medical facilities small and large. Integrated devices are poised to revolutionize everything from patient care to physician workload management to clinical trial design, but they lack a purpose‐built regulatory framework to both nurture and oversee their development. The US Food and Drug Administration (FDA) currently holds them to the same risk‐stratified premarket review standards as surgical instruments, x‐ray machines, and other analog tools, utilizing a regulatory framework first developed almost 50 years ago.^3^ As these integrated devices become increasingly more sophisticated and widespread, novel regulatory systems will be necessary to keep pace with their evolution.

Internet of things devices like AI‐augmented insulin pumps;^4^ fitness trackers that populate electronic health records (EHRs); bedside vital sign monitoring connected to nursing station terminals, mobile phones, and the EHR itself; gaze tracking systems; handheld ultrasound devices; and myriad mobile‐based health applications are already on the market,^5^ promising both real and significant clinical benefits. First, by personalizing services at the level of the individual, integrated devices stand to empower patients by offering them a greater level of control over their health. Second, they allow clinicians to operate at the top of their licenses by simplifying or automating data gathering or low‐level diagnostic decision making so that clinicians can focus on interpreting and guiding high‐level treatment, as is the case with automated vitals machines that feed data into various algorithms for sepsis or other potential causes of decompensation or with ultrasound machines that automatically calculate bladder volume or fetal gestational age. Third, these “smart” devices promote or facilitate automated, self‐service, and remote service medicine, thereby democratizing access and lowering costs, with a variety of devices offering consumers and patients the likes of automated/remote electrocardiograms (EKGs), auscultation, and physical therapy, among many more. On a macro level, they can enhance labor productivity in care delivery following over two decades of stagnation.^6^ Although the integration of new software and digital technologies has allowed for significant leaps and bounds forward, it has become abundantly clear that existing medical device regulatory frameworks were designed more to address the incremental advancements for which traditional, analog devices are better known. Unfortunately, there are few options for the paradigm‐changing devices to enter the market in a flexible fashion, which creates substantial barriers to entry and may even prevent an unknown number of novel devices from being developed in the first place. In contrast, there are a multitude of routes to approval for market entry for prescription drugs.^7^ This paper surveys existing FDA approaches to medical device oversight before outlining a model structure for review and regulation of integrated devices along with a flexible, stratified approach to assessing evidence of safety and effectiveness.

## The Next Generation of Medical Technology: Why Current FDA Regulatory Pathways Are Inadequate

**Figure 1:**
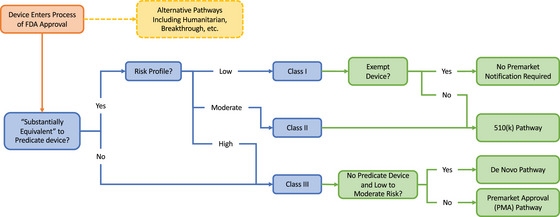
Existing Framework: FDA, Food and Drug Administration. [Colour figure can be viewed at wileyonlinelibrary.com]

Historically, the FDA's medical device regulatory frameworks were built with the aim of addressing both incremental and revolutionary innovation for traditional medical devices. The backbone of existing regulatory frameworks requires classification of traditional medical devices (Figure 1) based on the level of risk as class I (low risk), class II (moderate risk), or class III (high risk) devices.^8^, ^9^ The FDA then applies risk‐based regulatory interventions (e.g., class I devices are subject to general controls such as current good manufacturing practices, device registration and listing, and premarket notification, whereas class II devices require premarket notification and special controls such as meeting FDA‐recognized performance standards or being subject to postmarket surveillance). Class III devices must undergo a more comprehensive review process via the premarket approval (PMA) pathway akin to the new drug application review process for prescription drugs. New devices that do not have a predicate device are automatically classified as class III. The agency also practices risk‐based regulation in the context of device classification: if a new device is “substantially equivalent” to an existing approved class I or class II device, it can use the 510(k) clearance pathway, an abbreviated process intended for small, incremental changes.

In recognition of the difficulty posed by the statutory requirement of classifying new devices without predicate as high risk class III, policymakers created the De Novo review pathway,^10^ passed as part of the FDA Modernization Act of 1997, in order to allow low to moderate risk devices without a predicate device to be reclassified into lower risk regulatory classes and seek clearance via the 510(k) pathway instead of requiring the manufacturer to file a PMA. The PMA pathway presents a high threshold for approval, appropriate for revolutionary innovation, whereas the 510(k) pathway is purpose built for incremental innovation. The De Novo review pathway, among other exceptions, represents a compromise to expedite the review of lower risk devices. However, even with these limited flexibilities, the FDA has still been known to deem relatively minor changes as violative of “substantial equivalence,” triggering extensive rounds of testing, report generation, and filing to fulfill statutory standards.^11^, ^12^

Using these regulatory tools, the FDA has developed classifications for over 1,700 generic device types.^8^ At present, these classification decisions are made by the FDA's Office of Product Evaluation and Quality, housed within the Center for Devices and Radiological Health (CDRH), in which expert‐led review teams are grouped into 16 specialties (e.g., cardiovascular, orthopedic, etc.).^8^ However, these teams are largely siloed and lack central coordination for cross‐functional products such as those containing software. The FDA's Office of Combination Products (OCP), located outside the product centers in the Commissioner's office, is tasked with evaluating “combination products.” The OCP focuses on classification of a primary and secondary product area to drive cross‐center review for drug–device, drug–biologic, or biologic–drug products, with an integrated device such as software–hardware medical device combinations introducing a new layer of nuance thus far largely unaddressed by existing regulatory paradigms.

Integrated devices raise concerns over the limitations of the established medical device framework, which is designed around hardware with a single milestone barrier to market entry, a framework ill suited to both rapid cycle innovation and products comprised of multiple components. The FDA has attempted to adapt the existing framework to stand‐alone medical software products, referencing the International Medical Device Regulators Forum (IMDRF)’s risk‐weighted approach to defining “software as a medical device (SaMD).”^13^ Policymakers have attempted to assist this, with the 21st Century Cures Act establishing explicit statutory exceptions to the definition of an FDA‐regulated medical device for certain types of software, including software intended to be used for administrative support, encouraging healthy lifestyles, serving as electronic patient records, and supporting clinical laboratory data, with subsequent agency guidance clarifying additional software and hardware functions that would individually fall under this exception.^14^

Distinguishing garden‐variety medical devices from unregulated products under these exemptions can be difficult, as this strategy shifts the burden of line drawing onto manufacturers who self‐classify products in their submissions to the FDA, raising both the scope and stakes for regulatory circumnavigation.^15^ A prominent example of regulatory circumnavigation that resulted in dire consequences arose with Theranos, which took advantage of loopholes in the FDA's regulations regarding lab‐developed tests that were established over 40 years ago^16^ in addition to falsely labeling their test vials as class I devices when they should have been labeled as class II.^17^ Despite what turned out to be blatant fraud, Theranos's case highlights the high‐stakes strategic gamesmanship that can occur in order to circumnavigate regulations, with device classification being no exception as device manufacturers vie for favorable classifications that support their overall market strategy. The classification of these products is further complicated by the fact that many of these products feature components that span a number of different categories.

Take, for example, a hypothetical, AI‐enabled insulin pump that can interface with a smartphone to take photos of food, establish carbohydrate counts, and autoadjust insulin dosing in conjunction with a continuous glucose monitor. Given its novelty, would this combination of functions and intended uses require a PMA? Alternatively, do each of its distinct components (i.e., the insulin pump, the pump software, the phone app, and the continuous glucose monitor) require separate 510(k) filings? To what extent can the components be disaggregated? The regulatory uncertainty that these questions raise has real‐world consequences, as entrepreneurs and device manufacturers will find it hard to justify committing resources to developing integrated products that do not have clear paths to regulatory approval, ultimately delaying or resulting in a loss of access to innovative technologies as product development is abandoned or not even initiated in early stages. Indeed, the slow domestic development of automation in insulin pumps in conjunction with trials of open‐source models in New Zealand^18^ could be considered evidence of the need to update domestic FDA product regulation in order to reduce regulatory risk and spur domestic product innovation and testing.

The FDA has taken some measures to clarify this ambiguity, notably in its Multiple Function Products Guidance.^19^ This effectively treats software as distinct from the device under review, as designed by the manufacturer and as presented to the FDA in device hazard analysis. For example, the health applications such as the EKG function on an Apple iPhone would be considered distinct from its other, purely telecommunication, personalization, or entertainment functions, which fall outside the ambit of FDA review.^20^, ^21^ This has streamlined premarket review of SaMD operating on nonmedical device platforms, directly affecting safety and efficacy determinations, labeling, and the extent of required disclosures. However, the decoupling of software from the devices they run on represents only a partial solution to the larger question of integrated medical device product regulation. Although this distinction allows for some manufacturer‐driven separation of functional components, it only does so in the context of the determination of whether software is an FDA‐regulated medical device.

These questions become thornier still as device manufacturers partner with third‐party software providers to supply source code and implement solutions as well as when software products are capable of updating themselves in real‐time, like those employing ML or AI techniques to continually “learn” from new data. With this device universe representing over 500 devices, 75% of which are for radiological use,^22^ the need for a more purpose‐built regulatory review pathway could not be clearer. The agency has again attempted to adapt its current device regulatory framework, enumerating guidance for approval of *predetermined* change control plans for AI‐/ML‐enabled devices.^23^ Irrespective of the aforementioned, pulling apart these devices into fractional components clearly raises regulatory, contracting, ownership, and liability concerns, demonstrating that continued innovation necessitates an update of existing regulatory frameworks to provide greater regulatory certainty.

## International Approaches to Integrated Devices

The European Union (EU) has directed all relevant regulatory bodies to review combination products, including those supervising drug applications and standard device submissions, which resulted in the adoption of a three‐step regulatory paradigm. First, it established separate regimes for governing software (which are subject to the Medical Device Regulation [MDR]) and in vitro device (the In Vitro Diagnostic Medical Device Regulation [IVDR]) capabilities.^24^ If a candidate for regulatory review clearly falls into either category, then the relevant regulation (MDR versus IVDR) governs. If it does not, in the case of a true combination product, the EU then considers whether the software acts merely as an “accessory” (i.e., fulfilling an independent medical function) or as an integral “component” to the physical medical device. In making this determination, EU regulators look to the primary function of the device with an eye to the embedded software's location and function. If a traditional device function predominates, then it is subject to IVDR only, whereas if the “medical device software” function predominates, both IVDR and MDR apply. This is an enlightened approach, but it does suffer from some drawbacks. In some cases, hardware and software functions can be inextricably linked to the extent that many devices cannot operate at all without at least some of the software, and so, establishing a “primary” role may fail to capture a large portion of a product's functionality.

In contrast to the EU's functional disaggregation approach, a number of Asian jurisdictions have adopted an integrated approach to regulating combination products. South Korea's Ministry of Food and Drug Safety has created a purpose‐built framework for software products, regardless of their role in the device.^25^ It does so by following a risk‐stratified paradigm that borrows heavily from IMDRF's approach to SaMD.^26^ The combined hardware–software product is subject to premarket review in its entirety, thereby obviating the need for tortuous regulatory line drawing. Similarly, in Japan, devices are submitted to a central regulator, the Pharmaceuticals and Medical Devices Agency, which issues a single device classification for an integrated combination product.^27^, ^28^

## New Directions: A Voluntary Pathway for Integrated Devices

**Figure 2:**
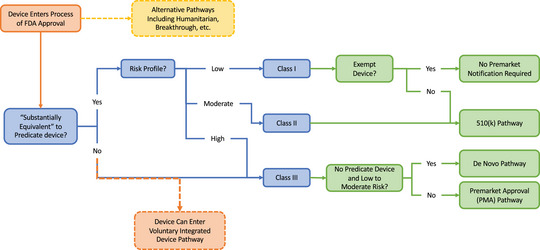
New, Voluntary‐Integrated Device Pathway: FDA, Food and Drug Administration. [Colour figure can be viewed at wileyonlinelibrary.com]

**Figure 3:**
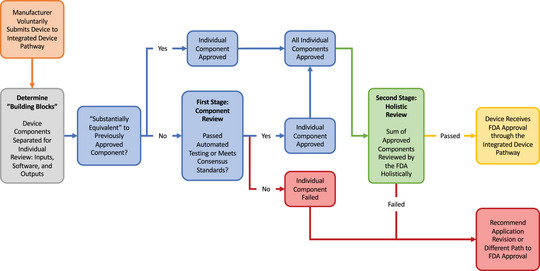
Integrated Device Pathway: FDA, Food and Drug Administration. [Colour figure can be viewed at wileyonlinelibrary.com]

Although the United States's risk‐based regulatory framework is well calibrated to promote incremental and revolutionary innovation for traditional medical devices, a voluntary alternative pathway to support revolutionary innovation for cross‐functional products, specifically in regard to integrated devices, is sorely lacking. Integrated devices should be offered an alternative, voluntary pathway for FDA review (Figure 2) that can be utilized by any CDRH divisional office and takes into consideration the interplay of software and hardware components. The proposed use of a voluntary pathway is in recognition of the fact that there is great variability in medical devices currently, with inevitably more variability in the future, so creating versatile and flexible pathways to accommodate new and unique devices will be important moving forward. Today, nearly all electronic devices feature some built‐in software, and the sheer ubiquity of clinically relevant code renders this an increasingly important area. Under this model, we propose that a product undergo two‐stage review, with the first stage composed of independent component review, and the second stage would be a holistic review (Figure 3). During the first stage, input, software, and device output components would be identified and tested independently from one another.

Inputs would be defined as any trigger or data—which could take the form of a stimulus passed through a built‐in sensor, data shared from a different device, training data for an ML product, or user‐entered input—that is used to initiate the software function of the device. The reliability, reproducibility, and relevance of inbound data should be verified to ensure that the software built into the device is operating off of high‐fidelity inputs to prevent misfires or errant outputs. In practice this would simply mean ensuring that sensors and other inputs are tested to ensure that they are functioning and performing as expected. This input evaluation should also include special considerations for those devices that are consumer, clinician, or some other human facing, as user error is a real threat to patient safety that can be substantially mitigated with the appropriate use of user interface and user experience design controls.

The software components are those components that interpret the input in order to produce an intended function. These functions vary widely, with examples including signal‐related functions (e.g., translating the analog sensor signal to a digital signal), control functions (e.g., activating a valve or switching on heating coils), device management functions (e.g., remote device configuration, fault monitoring, user interactions), and network functions (e.g., transmitting data to and from network controllers). Typically, these components are the software that run behind the scenes and dictate the functions of most devices, often hidden behind a graphical user interface that makes it easier for users to interact with the device. Although these functions are integrated within one device, a well‐designed device should allow testing of each function separately, which would also ease regulatory burden, as manufacturers would be able to reuse software components (e.g., user interface, diagnostics, update, and network functions) across their devices without having to subject each instance/device to review unless there are substantial modifications.

Regulation of software components that utilize AI and ML is a particularly pressing issue, with the rapid development of those technologies already being built into health care applications. One would expect that AI and ML will initially be deployed in lower risk use cases, perhaps simply to enhance or augment safety in provider‐ and consumer‐facing devices. These technologies will eventually reach broad penetration throughout the health care ecosystem, likely even operating autonomously. Although it is difficult to predict the exact trajectory of AI and ML technologies and their specific future health care use cases, they must still be predictable enough to submit for review in a meaningful way. However, the specifics of that review process are beyond the scope of this paper.

The last piece to be reviewed would be the device output components, which would be any component that acts on the environment or patient or otherwise produces an effect external to the device. This effect would be the resultant function of the software component whether that is a test result readout, delivering a drug, changing ventilator or other device settings, feeding data into a different device, etc. This would require testing the consistency with which these integrated devices, given the correct command functions, produce the intended output or result. Although the fidelity of the input and the software are both important links in this chain, the output is what ultimately reaches the patient, which merits particular care. Again, these output components should be able to be evaluated as distinct components, allowing for manufacturers to also reuse these components as well.

The piecemeal strategy of this framework means that manufacturers would not necessarily need the FDA to develop classifications for each new iteration of a category of devices. Instead, manufacturers could submit individual components for review, providing the FDA with previously authorized “building blocks.” The sum of these “building blocks” would then undergo the second stage of review, or the holistic review, and this two‐stage framework would improve regulatory efficiency and decrease burdens on manufacturers. This would open up a whole new world of possibilities, as manufacturers could develop increasingly more complex and sophisticated implementations of their devices—featuring cutting edge software components—without having to worry about fitting their whole device into a preexisting classification. Establishing a purpose‐built regulatory scheme in this way would mark a step toward modernizing regulatory oversight, stimulate innovation, and bring the United States in line with international standards.

## Pathways to Meeting the Evidentiary Burden

As with drugs and standard medical devices, an integrated device must meet the FDA's evidentiary burden of safety and efficacy, with the sponsor bearing the burden of providing the appropriate evidence. The first stage of review would rely on product “building blocks,” with manufacturers meeting the evidentiary burden in a flexible fashion through compliance with consensus standards, automated testing, third‐party review, or another process. The FDA already supports the development of consensus standards through its Standards and Conformity Assessment Program,^29^ through which manufacturers are currently able to submit “Declarations of Conformity” for certain industry‐created standards that the FDA has recognized.^30^ Automated or manual function tests, which are routinely performed during manufacture and quality control, are another alternative to meeting the evidentiary burden for device components and would be examined by FDA inspectors. Automated or manual function tests would ensure that input signals are appropriately detected, data integrity is preserved throughout the functioning of these devices, and safe operating bounds are built into these devices, among other safety and quality metrics. The synthesis of submitted data and external validation would follow the pattern established by the Federal Communications Commission (FCC)’s approach to approving telecommunications products. In addition to relying on manufacturers’ “Declarations of Conformity,” the FCC also performs its own tests to “certify” products meeting a higher standard of technical specifications.^31^, ^32^

However, there are a number of ways to help lessen this burden both to safeguard high quality and safety standards as well as to encourage continued innovation. In conjunction with the standard criteria of device review ordinarily overseen by CDRH, the FDA could establish a set of common standards for automated testing, perhaps even offering expedited review to submissions adhering to them. By promoting best practices in software development through automated testing, the FDA could ease the review process for both the FDA and sponsors alike by not only reducing the resources required to complete reviews but also providing substantial regulatory predictability to help offload the risk that manufacturers take on when designing and producing new products.

Furthermore, manufacturers can more easily surmount the evidentiary burden by referencing prior approvals of existing hardware or software components [e.g., 510(k) predicates or existing PMAs], whereas the FDA should apply principles of risk‐based oversight for completely new devices or for those featuring substantially revised components to ensure that review is not more burdensome than preexisting review pathways. Lastly, the FDA should artfully distribute pre‐ and postmarket risk by following a total product life cycle approach to integrated device regulation. This could include codifying and extending certain elements of the Digital Health Pre‐Certification Program, such as its automated reporting features (although this pilot as a whole was less than successful),^33^ which would dovetail with the use of standardized automated testing to reduce the burden of postmarket surveillance.

In an effort to further support common standards for automated testing, the FDA or a designated third party can furnish manufacturers with publicly available test data sets that can be used for (1) validation at each step, and (2) evaluation prior to entering the regulatory pathway, further promoting regulatory certainty and reducing risk. Additionally, test data sets could be built on accumulated experience and faults, functionally allowing for “spell‐checking” developers in the field who may otherwise waste valuable time identifying—or worse yet, missing altogether—frequently encountered design pitfalls and catalyzing the overall development process. This strategy would be in line with what other federal government agencies are aiming to do, such as the National Institute of Standards and Technology, which has developed a common set of evaluations for facial recognition technology.^34^ Funding for the development of these data sets could be drawn from the National Institutes of Health, which have earmarked funds to support the development of a library of training data sets for AI technologies.^35^

Some policy analysts may raise concerns that the first phase of integrated device review would seem like industry self‐regulation; however, these concerns would be misplaced, as developing automated testing and consensus‐driven standards would include relevant stakeholders throughout industry and result in the creation of a mutually agreed‐on liability safe haven that would be acceptable to both the private sector—who would be compelled to participate to preserve their own self‐interest—as well as the FDA, who would ultimately decide whether to recognize any new standards proposed by the private sector. Although the first phase of review focuses on deterministic components, the second phase of review is nondeterministic, focusing on the user, use, and device performance/self‐regulation along with emergent properties, including human factors or human interface questions that cannot undergo automated evaluation. Some devices can self‐regulate their own use and can be used by a wide range of operators (e.g., a registered nurse in a community health center), whereas others require a trained operator (e.g., a surgeon who has completed a single procedure thousands of times). The second phase or holistic review stage will consider these questions, enabling the FDA to efficiently deploy its technical human capital at the appropriate stage of review. In this way, this voluntary pathway would not be any less rigorous than existing pathways; rather, it would be more tailored for the unique needs of integrated devices and borrow from frameworks used by other agencies, such as the FCC, with the use of Declarations of Conformity and certification of products using their own testing as previously mentioned.

Some stakeholders may be averse to changes in FDA regulatory policy. Some larger, established medical device manufacturers may prefer the existing regulatory system, viewing an alternative pathway as a threat that would increase competition. In contrast, other large manufacturers may see this as an opportunity to build new lines of business, whereas technology entrepreneurs likely would support improvement in FDA product regulation, allowing them to use their combined knowledge of software and hardware engineering to improve the patient experience and outcomes and lower costs by changing clinical operations. Finally, sectors of physicians may be uncomfortable with the democratization and potential automation of medical services provisioned by integrated devices, noting that the use of advanced technology may eventually become the standard of care.

From an organizational standpoint, the proposed integrated device review pathway should be another arrow in CDRH's regulatory quiver to more systematically and comprehensively evaluate integrated devices without taking away from CDRH's current structure of categorizing products by intended use. This would, however, likely require the hiring of substantial technical digital health talent to support the implementation of this regulatory framework, which could be accomplished via special hiring and pay authorities that allow for the recruitment and hiring of physicians (Title 38 of the Cures Act)^36^ and technical experts or “special consultants” [Title 42(f) of the Cures Act].^36^ Critically, one would expect that operating costs would eventually diminish at scale as the process becomes progressively more automated, which is particularly important, as this program would likely require a large infusion of funds up front to initiate but would be sustained over the long term through application user fees paid by those companies electing to use this pathway. Additionally, the FDA recently established a standing Digital Health Advisory Committee,^37^ facilitating selective external engagement of experts when higher‐risk products such as those involving more autonomous AI and ML capabilities are on the threshold of approval and the FDA has specific technical questions requiring external feedback. Although the committee description does not specify domains of expertise,^38^ ideally, the committee should feature representatives from provider organizations (specifically those who are currently actively practicing), academic research centers, device manufacturers, software developers, and cybersecurity researchers in order to synthesize input from all relevant stakeholders.

## Conclusion

As integrated medical devices grow in sophistication and prevalence, a new voluntary, alternative regulatory pathway is needed if the FDA is to keep pace with and surpass industry. Establishing a dedicated voluntary alternative pathway for integrated devices would bring the FDA's review processes in line with contemporary technical developments while also allowing for greater flexibility in meeting regulatory requirements for both manufacturers and regulators. Evaluation of each component of integrated devices separately ensures that this framework can evolve to accommodate drastic shifts in future technologies, as it does not require creating specific classifications for each individual device type. Furthermore, components would serve as building blocks of devices undergoing holistic review by agency reviewers, thus effectively deploying FDA human capital more efficiently to focus on nondeterministic device review. Through creation of this new review pathway, policymakers would build on decades of fragmented regulatory regimes and pilot projects, drawing on their best features and coalescing them within a dedicated structure. Congress should provide the FDA with new legislative authority, establishing such an approach as outlined here that would be supplemental to, and not replace, the established risk‐based medical device regulatory framework. Although much of health regulatory programs are stuck in an analog world, the marketplace is moving to a technology‐driven health care system. It's time for regulatory policy to catch up.

## Funding/Support

Drs. Miller and Cho report funding from the Charles Koch Foundation, which had no role in this work.

## Conflict of Interest Disclosures

Dr. Gowda reports receiving fees outside the related work from Give Legacy, Inc., a fertility preservation startup. Dr. Cho reports prior service as a fellow at the Centers for Medicare and Medicaid Services. Dr. Schulzrinne worked at the FCC from 2010 through 2017, including as Chief Technology Officer from 2012 to 2014 and in 2017. Dr. Miller previously served as a Senior Policy Fellow for Health Information Technology at the FCC in 2015 and as a medical officer at the FDA from 2016 to 2017. He reports prior service as a member of the Medicare Evidence Development and Coverage Advisory Committee, Centers for Medicare and Medicaid Services, and current service as a Commissioner on the Medicare Payment Advisory Commission and receiving fees outside the related work from the Federal Trade Commission, the Maryland Neurosurgical Society, the California Association of Neurosurgeons, and the Digestive Health Physicians Association. He also reports grant support outside the related work from Arnold Ventures and Ohio State University.

## References

[1] Connected Medical Device Market ‐ Growth, Trends, COVID‐19 Impact, and Forecasts (2022 ‐ 2027) . Mordor Intelligence. 2022. Accessed June 10, 2023. https://www.mordorintelligence.com/industry‐reports/connected‐medical‐device‐market

[2] Healthcare Connected Devices Market: Current Analysis and Forecast (2020‐2027) . UnivDatos Market Insights. 2022. Accessed June 10, 2023. https://univdatos.com/report/healthcare‐connected‐devices‐market/

[3] Medical Device Regulation Act , PL 94–295, 94th Cong (1975‐1976) 1976.

[4] Nimri R , Battelino T , Laffel LM , et al. Insulin dose optimization using an automated artificial intelligence‐based decision support system in youths with type 1 diabetes. Nat Med. 2020;26(9):1380‐1384. 10.1038/s41591-020-1045-7 32908282

[5] National Research Council Committee on the Role of Human Factors in Home Health Care . The Role of Human Factors in Home Health Care: Workshop Summary. National Academies Press; 2010. 24983032

[6] Private community hospitals labor productivity . US Bureau of Labor Statistics. Updated June 30, 2022. Accessed June 6, 2023. https://www.bls.gov/productivity/highlights/hospitals‐labor‐productivity.htm

[7] Drug Approval Pathways. American Academy of Neurology. 2020. Accessed June 10, 2023. https://www.aan.com/siteassets/home‐page/policy‐and‐guidelines/policy/priority‐issues/drug‐pricing/drug‐approval‐pathways.pdf

[8] Sarata AK . FDA Regulation of Medical Devices. Congressional Research Service. 2023. Accessed June 10, 2023. https://crsreports.congress.gov/product/pdf/R/R47374

[9] Darrow JJ , Avorn J , Kesselheim AS . FDA regulation and approval of medical devices: 1976–2020. JAMA. 2021;326(5):420‐432. 10.1001/jama.2021.11171 34342614

[10] Medical Device De Novo Classification Process , 86 FR 54826, 21 CFR §860. 2021.

[11] Kadakia KT , Beckman AL , Ross JS , Krumholz HM . Renewing the call for reforms to medical device safety—the case of penumbra. JAMA Intern Med. 2022;182(1):59‐65. 10.1001/jamainternmed.2021.6626 34842892

[12] Institute of Medicine ; Board on Population Health and Public Health Practice ; Committee on the Public Health Effectiveness of the Food and Drug Administration 510(k) Clearance Process . Medical Devices and the Public's Health: The FDA 510(k) Clearance Process at 35 Years. National Academies Press; 2011.

[13] Software as a Medical Device Working Group . Software as a Medical Device (SAMD): Clinical Evaluation: Guidance for Industry and Food and Drug Administration Staff. US Food and Drug Administration; 2017. Accessed June 10, 2023. https://www.fda.gov/regulatory‐information/search‐fda‐guidance‐documents/software‐medical‐device‐samd‐clinical‐evaluation

[14] Medical Device Data Systems . Medical Image Storage Devices, and Medical Image Communications Devices: Guidance for Industry and Food and Drug Administration Staff. US Food and Drug Administration; 2022. Accessed March 15, 2023. https://www.fda.gov/regulatory‐information/search‐fda‐guidance‐documents/medical‐device‐data‐systems‐medical‐image‐storage‐devices‐and‐medical‐image‐communications‐devices

[15] Devereaux A . Approaching the singularity behind the veil of incomputability: on algorithmic governance, the economist‐as‐expert, and the piecemeal circumnavigation of the administrative state. SSRN. 2019. 10.2139/ssrn.3332798

[16] Parkins K . The Theranos saga: a wake‐up call for the lab‐developed test market. Medical Device Network. January 25, 2022. Accessed June 10, 2023. https://www.medicaldevice‐network.com/features/theranos‐ldt‐regulation/

[17] Rutschman AS . How Theranos’ faulty blood tests got to market – and what that shows about gaps in FDA regulation. The Conversation. October 5, 2021. Accessed June 10, 2023. https://theconversation.com/how‐theranos‐faulty‐blood‐tests‐got‐to‐market‐and‐what‐that‐shows‐about‐gaps‐in‐fda‐regulation‐168050

[18] Burnside MJ , Lewis DM , Crocket HR , et al. Open‐source automated insulin delivery in type 1 diabetes. N Engl J Med. 2022;387(10):869‐881. 10.1056/NEJMoa2203913 36069869

[19] Multiple Function Device Products: Policy and Considerations. US Food and Drug Administration; 2020. Accessed June 10, 2023. https://www.fda.gov/regulatory‐information/search‐fda‐guidance‐documents/multiple‐function‐device‐products‐policy‐and‐considerations

[20] Krueger AC . Review of De Novo request for classification of the ECG App (Apple). US Food & Drug Administration; 2018. Accessed June 10, 2023. https://www.accessdata.fda.gov/cdrh_docs/pdf18/den180044.pdf

[21] Expanded Use of Apple ECG App for Supporting Remote Heart Rhythm Evaluation During the COVID‐19 Pandemic. Apple; 2020. Accessed June 10, 2023. https://www.apple.com/healthcare/docs/site/Apple_ECG_app_during_COVID‐19.pdf

[22] Artificial intelligence and machine learning (AI/ML)‐enabled medical devices. US Food and Drug Administration. Updated October 5, 2022. Accessed June 4, 2023. https://www.fda.gov/medical‐devices/software‐medical‐device‐samd/artificial‐intelligence‐and‐machine‐learning‐aiml‐enabled‐medical‐devices

[23] Marketing Submission Recommendations for a Predetermined Change Control Plan for Artificial Intelligence/Machine Learning (AI/ML)‐Enabled Device Software Functions: Draft Guidance for Industry and Food and Drug Administration Staff. US Food and Drug Administration; 2023. Accessed June 10, 2023. https://www.fda.gov/media/166704/download

[24] Baird P , Cobbaert K . Software as a Medical Device: A Comparison of the EU's Approach With the US's Approach . British Standards Institution. *Medical Device White Paper Series*. Accessed June 10, 2023. https://www.bsigroup.com/globalassets/localfiles/en‐th/Medical%20devices/brochure/software‐as‐a‐medical‐device–th.pdf

[25] Yang J‐Y . Regulatory Updates on Medical Devices in Korea. Ministry of Food and Drug Safety; 2019. Accessed June 10, 2023. https://www.imdrf.org/sites/default/files/docs/imdrf/final/meetings/imdrf‐meet‐190916‐russia‐yekaterinburg‐24.pdf

[26] Baik S . South Korea releases guidance for software using big data, AI, and machine learning. Asia Actual. September 18, 2022. Accessed June 10, 2023. https://asiaactual.com/blog/south‐korea‐software‐guidance‐big‐data‐ai‐machine‐learning/

[27] Tian J , Song X , Wang Y , et al. Regulatory perspectives of combination products. Bioact Mater. 2022;10:492‐503. 10.1016/j.bioactmat.2021.09.002 34901562 PMC8637005

[28] Gropp M . Combination products. Presented at: The 2006 Symposium of APEC Network on Pharmaceutical Regulatory Science; October 12–13, 2006; Tokyo, Japan. Accessed June 10, 2023. https://www.pmda.go.jp/files/000150384.pdf

[29] Standards and Conformity Assessment Program. US Food and Drug Administration. Updated September 19, 2023. Accessed June 10, 2023. https://www.fda.gov/medical‐devices/premarket‐submissions‐selecting‐and‐preparing‐correct‐submission/standards‐and‐conformity‐assessment‐program

[30] Recognized consensus standards: medical devices. US Food and Drug Administration. Updated December 18, 2023. Accessed June 10, 2023. https://www.accessdata.fda.gov/scripts/cdrh/cfdocs/cfstandards/search.cfm

[31] Equipment authorization procedures. Federal Communications Commission. Accessed March 16, 2023. https://www.fcc.gov/general/equipment‐authorization‐procedures‐0

[32] Equipment authorization approval guide. Federal Communications Commission. Accessed March 16, 2023. https://www.fcc.gov/engineering‐technology/laboratory‐division/general/equipment‐authorization

[33] The Software Precertification (Pre‐Cert) Pilot Program: Tailored Total Product Lifecycle Approaches and Key Findings. US Food and Drug Administration; 2022. Accessed June 10, 2023. https://www.fda.gov/media/161815/download

[34] Grother P , Ngan M , Hanaoka K . Face Recognition Vendor Test Ongoing: General Evaluation Specifications. National Institute of Standards and Technology, US Department of Commerce; 2023. Accessed June 10, 2023. https://pages.nist.gov/frvt/api/FRVT_common.pdf

[35] Bridge to Artificial Intelligence (Bridge2AI) . National Institutes of Health. Updated November 21, 2023. Accessed March 16, 2023. https://commonfund.nih.gov/bridge2ai

[36] FDA 21st Century Cures Workforce Planning: Report to Congress. US Food and Drug Administration; 2018. Accessed June 10, 2023. https://www.fda.gov/media/114163/download

[37] FDA establishes new advisory committee on digital health technologies. US Food and Drug Administration. Updated October 12, 2023. Accessed June 10, 2023. https://www.fda.gov/news‐events/press‐announcements/fda‐establishes‐new‐advisory‐committee‐digital‐health‐technologies

[38] Swink J . FDA digital health advisory committee. US Food and Drug Administration. Updated January 8, 2024. Accessed June 10, 2023. https://www.fda.gov/medical‐devices/digital‐health‐center‐excellence/fda‐digital‐health‐advisory‐committee

